# Role of the KATP channel in the protective effect of nicorandil on cyclophosphamide-induced lung and testicular toxicity in rats

**DOI:** 10.1038/srep14043

**Published:** 2015-09-25

**Authors:** Lamiaa A. Ahmed, Shohda A. EL-Maraghy, Sherine M. Rizk

**Affiliations:** 1 Department of Pharmacology and Toxicology, Faculty of Pharmacy, Cairo University, Cairo, Egypt; 2 Department of Biochemistry, Faculty of Pharmacy, Cairo University, Cairo, Egypt

## Abstract

This study is the first to investigate the role of the KATP channel in the possible protection mediated by nicorandil against cyclophosphamide-induced lung and testicular toxicity in rats. Animals received cyclophosphamide (150 mg/kg/day, i.p.) for 2 consecutive days and then were untreated for the following 5 days. Nicorandil (3 mg/kg/day, p.o.) was administered starting from the day of cyclophosphamide injection with or without glibenclamide (5 mg/kg/day, p.o.). Nicorandil administration significantly reduced the cyclophosphamide-induced deterioration of testicular function, as demonstrated by increases in the level of serum testosterone and the activities of the testicular 3β- hydroxysteroid, 17β-hydroxysteroid and sorbitol dehydrogenases. Furthermore, nicorandil significantly alleviated oxidative stress (as determined by lipid peroxides and reduced glutathione levels and total antioxidant capacity), as well as inflammatory markers (tumour necrosis factor-α and interleukin-1β), in bronchoalveolar lavage fluid and testicular tissue. Finally, the therapy decreased the levels of fibrogenic markers (transforming growth factor-β and hydroxyproline) and ameliorated the histological alterations (as assessed by lung fibrosis grading and testicular Johnsen scores). The co-administration of glibenclamide (a KATP channel blocker) blocked the protective effects of nicorandil. In conclusion, KATP channel activation plays an important role in the protective effect of nicorandil against cyclophosphamide-induced lung and testicular toxicity.

Cyclophosphamide (CP) is a cytotoxic alkylating agent that is widely used either as an antineoplastic or an immunosuppressant. CP is used for the treatment of leukaemia, multiple myeloma, lymphomas and rheumatoid arthritis, as well as in preparation for bone marrow transplantation^1^,^2^. However, multiple organ toxicity, including lung and testicular toxicity, is a major problem that limits its optimal use in both experimental and clinical studies^3^,^4^,^5^. The antineoplastic effects of CP are mainly related to its metabolite phosphoramide mustard, whereas its toxic side effects, such as cell death (apoptosis, oncosis and necrosis), are linked to acrolein, the principal toxic metabolite of CP^6^,^7^.

Oxidative stress plays a pivotal role as one of the major mechanisms involved in the pathophysiology of CP toxicity. Acrolein interferes with the tissue antioxidant defence system and results in the production of highly reactive oxygen species^8^,^9^. Free radicals can induce lipid peroxidation of structural membranes and interact with other biomolecules, such as enzymes, receptors and ion pumps, causing inactivation or inhibition of their normal functions^10^. Oxidative stress-mediated disruption of the redox balance generates biochemical and physiological alterations, with the activation of several intracellular signalling pathways leading to the upregulation of proinflammatory cytokine synthesis and the induction of apoptosis^11^. Moreover, chronic unresolved inflammation plays a major role in pulmonary fibrogenesis and testicular toxicity^12^. Additionally, decreased collagen degradation and increased collagen biosynthesis may be key features of the maintenance and progression of fibrosis, where the inhibition of proteases that degrade the extracellular matrix also modulates profibrogenic and proinflammatory cytokines, such as transforming growth factor-β (TGF-β) and tumour necrosis factor-α (TNF-α)^12^,^13^.

Although the biochemical and histopathological events associated with CP toxicity have been well studied, effective therapeutic interventions remain unavailable. Nicorandil is an anti-anginal agent that acts not only as a KATP channel opener but also as a nitric oxide (NO) donor. The major beneficial effects of nicorandil are attributed to its KATP channel-opening property, while its NO donating property functions adjunctively in this setting^14^. Nicorandil has previously been shown to inhibit the development of experimentally induced prostatic hyperplasia and mesangioproliferative glomerulonephritis^15^,^16^. Nicorandil, as a KATP channel opener, has also been shown to inhibit monocrotaline-induced inflammation and proliferation in lung tissue in rats^17^. Importantly, the administration of nicorandil has been found to reduce oxidative stress by stimulating KATP channel opening, independently of its ability to donate NO^18^. Therefore, the use of nicorandil may be an appropriate approach to reducing CP-induced toxicity.

The aim of the present study was to investigate the protective effect of nicorandil on CP-induced lung and testicular toxicity. It also examined the role of KATP channel opening in the possible protection mediated by nicorandil.

## Material and Methods

### Animals

Male Wistar rats weighing 170–190 g were obtained from the animal facility of the Faculty of Pharmacy, Cairo University, Egypt. The animals were housed under controlled environmental conditions at constant temperature (25 ± 2 °C) and a 12/12 h light/dark cycle. The rats were allowed standard chow diet and water ad libitum. The investigation complied with the *Guide for Care and Use of Laboratory Animals* published by the US National Institutes of Health (NIH Publication No. 85-23, revised 2011) and was approved by the Ethics Committee for Animal Experimentation at the Faculty of Pharmacy, Cairo University (Permit Number: PT 1231).

### Chemicals

CP was purchased from Baxter Oncology (Frankfurt, Germany). Nicorandil and glibenclamide were obtained from Adwia Pharmaceutical Company (Cairo, Egypt) and Pharco Pharmaceutical Company (Alexandria, Egypt), respectively. Fine chemicals and reagents, unless otherwise specified, were obtained from Sigma-Aldrich Chemical Co. (St. Louis, MO, USA).

### Experimental Design

The rats were randomly divided into four groups. Group I (n = 8) served as the normal group. Group II (n = 10) received CP dissolved in normal saline (150 mg/kg/day, i.p.) for 2 consecutive days followed by 5 days of no treatment^19^. Group III (n = 8) received CP as in Group II and nicorandil (3 mg/kg/day, p.o.) starting from the day of CP injection until the end of the experiment^20^. Group IV (n = 10) was given the same regimen as Group III together with glibenclamide (5 mg/kg/day, p.o.) 2 h before nicorandil administration^14^. Notably, rats treated with CP and glibenclamide only showed similar results to that observed in Group II (data not shown). At the end of the experimental period, the rats were weighed and anaesthetized for blood sampling collection from the retro-orbital sinus. The animals were then euthanized, the thoracic cavity was exposed and a needle was inserted in the right ventricle to wash the lung with ice-cold physiological saline. For bronchoalveolar lavage fluid (BALF) collection, the trachea was cannulated, and the lung was lavaged with ice-cold sterile physiological saline three times with a volume of 3 ml/wash. Whole lungs and testes were then rapidly excised, washed with ice-cold saline, dried and weighed.

### Biochemical measurements

BALF data represent whole lung status and have commonly been used for monitoring therapeutic efficacy and toxicity in laboratory animals. Therefore, biochemical indices of lung toxicity have been estimated mostly using BALF in the present study. BALF was centrifuged after collection at 600 xg for 10 min to sediment the cell debris prior to conducting biochemical assays. Portions of the right testis and right lung were also homogenized in ice-cold saline to prepare a 10% homogenate for biochemical measurements. The other portion of the same testis was used for western blot analysis, whereas the left testis and left lung were used for histological examination. The total protein content in BALF and testicular homogenates was determined according to the method of Lowry *et al.*^21^

### Serum testosterone

The serum testosterone level was assayed using an automated chemiluminescence immunoassay system (ADVIA Centaur; Bayer Vital, Fernwald, Germany). The procedure was performed according to the manufacturer’s instructions. The results were expressed as ng/dl.

### Testicular 3β- and 17β-hydroxysteroid dehydrogenase (HSD) and sorbitol dehydrogenase (SDH) activities

Testicular 3β-HSD and 17β-HSD activities were measured according to the methods of Talalay^22^ and Jarabak *et al.*^23^ 3β-HSD activity was measured after the addition of NAD^+^ to the tissue incubation mixture containing dehydroepiandrosterone (dissolved in ethanol) in sodium pyrophosphate buffer (pH 8.9). The increase in NADH absorbance was followed at 340 nm, and the results are expressed as nmol NADH/min/mg protein. 17β-HSD enzyme activity was measured after the addition of NADP^+^ to the tissue incubation mixture containing bovine albumin and testosterone in sodium pyrophosphate buffer (pH 10.2). The increase in absorbance was measured at 340 nm, and the results are expressed as nmol NADPH/min/mg protein. SDH activity was determined by the method of Chauncey *et al.*^24^, which depends on the conversion of D-sorbitol into D-fructose in the presence of NAD^+^. The increase in NADH absorbance was assessed at 340 nm for 3 min. One unit of SDH is defined as the amount of enzyme that catalyses the formation of 1 μmol NADH per min at 37 °C. The results are expressed as U/mg protein.

### Lipid peroxidation products

The total amount of lipid peroxidation in BALF and testicular tissue is indicated by the thiobarbituric acid reactive substance (TBARS) content, as described by Buege and Aust^25^. This method depends on the colorimetric determination of a pink pigment product resulting from the reaction of TBARS with thiobarbituric acid in acidic medium at high temperature. The resultant coloured product was measured at 535 nm, and the results are expressed as nmol/mg protein.

### Reduced glutathione (GSH)

BALF and testicular GSH levels were determined using the method of Beutler *et al.*^26^, where GSH reduces Ellman’s reagent [5,5’-dithiobis (2-nitrobenzoic acid)] (DTNB) to form a stable yellow product (5-mercapto-2-nitrobenzoic acid), which can be measured colorimetrically at 412 nm. The results are expressed as μmol/mg protein.

### Total antioxidant capacity (TAC)

BALF and testicular TAC were assessed using a colorimetric kit purchased from Bio-Diagnostic, Dokki, Giza, Egypt. The procedure was performed according to the manufacturer’s instructions. The results are expressed as μmol/mg protein.

### Tumour necrosis factor-α (TNF-α) and interleukin-1β (IL-1β)

TNF-α and IL-1β levels were estimated in BALF and testes using rat ELISA kits supplied by ID Labs Biotechnology Inc., Canada and BioVendor Research and Diagnostic Products, Candler, NC, USA, respectively. The assays were performed according to the manufacturer’s instructions, and the results are expressed as pg/mg protein for TNF-α level and μg/mg protein for IL-1β level.

### N-acetyl-β-D-glucosaminidase (NAG)

BALF NAG activity was assayed using the colorimetric method of Horak *et al.*^27^ This method was based on enzymatic hydrolysis of the substrate 4-nitrophenyl N-acetyl-β-D-glucosaminide by NAG in citrate buffer (pH 4.4) at 37 °C within 15 min. The liberated p-nitrophenol produced a yellow colour in alkaline medium which was measured at 405 nm, and the results are expressed as μmol p-nitrophenol/min/mg protein.

### Histamine and leukotriene C4 (LTC4)

The histamine and LTC4 levels were assayed in BALF using rat ELISA kits purchased from DIAsource ImmunoAssays (Louvain-la-Neuve, Belgium) and Neogen Corporation (Lansing, MI, USA), respectively. The assays were performed according to the manufacturer’s instructions, and the results are expressed as μg/mg protein.

### Transforming growth factor-β (TGF-β)

The TGF-β level was assayed in BALF using a rat ELISA kit purchased from WKEA Med Supplies Corp., China. The assays were performed according to the manufacturer’s instructions, and the results are expressed as ng/mg protein. To assay testicular TGF-β protein expression, a portion of the right testis was suspended in lysis buffer, and the protein levels were quantified using a Bio-Rad protein assay kit, USA. Testicular TGF-β1 protein expression was assessed as previously described^28^ using anti-TGF-β1 antibody (Santa Cruz Biotechnology Inc., Santa Cruz, CA, USA). Chemiluminescence detection was performed with the Amersham detection kit according to the manufacturer’s protocols and exposure to X-ray film. The amount of TGF-β1 protein was quantified by densitometric analysis of the autoradiograms using a scanning laser densitometer (Biomed Instrument Inc., USA). The results are expressed as arbitrary units after normalization to beta-actin (β-actin) protein expression.

### Hydroxyproline

The hydroxyproline content was determined according to the method described by Woessner^29^. Lung and testicular specimens were hydrolysed in 6 N HCl at 100 °C for 24 h. After neutralization, hydroxyproline was oxidized by buffered chloramine T reagent at room temperature. The chromophore was developed by heating with Ehrlich’s reagent (p-dimethylaminobenzaldehyde) at 60 °C and was detected at 555 nm. The results are expressed as μg/g wet tissue.

### Histological examinations

For light microscopic examination, the left lung was isolated, rinsed in ice-cold saline and immediately fixed in 10% formalin for 24 h. The specimens were processed for paraffin embedding, and 5 μm sections were prepared. The sections were stained with Masson’s trichrome and examined by microscopy (magnification x100). The images were captured and processed using Adobe Photoshop (version 8.0). The images were analysed by two investigators who were blinded to the type of treatment, and the severity of fibrosis was assessed according to the method proposed by Ashcroft *et al.*^30^. Briefly, the grade of lung fibrosis was scored on a scale from 0 to 8 by examining 5 randomly selected fields per section, and the average score was calculated. The criteria for grading lung fibrosis were as follows: grade 0, normal lung; grade 1, minimal fibrous thickening of the alveolar or bronchiolar walls; grade 3, moderate thickening of the walls without obvious damage to the lung architecture; grade 5, increased fibrosis with definite damage to the lung structure and with the formation of fibrous bands or small fibrous masses; grade 7, severe distortion of the lung structure with large fibrous areas; and grade 8, total fibrous obliteration of the fields. Similarly, the left testis was fixed in Bouin solution for 24 h, and 5 μm sections were prepared. The sections were then stained with haematoxylin and eosin and examined using a light microscope (magnification x100). To evaluate spermatogenesis, seminiferous tubules were scored by means of the Johnsen score as described by Johnsen^31^ on a scale from 1 (no seminiferous epithelial cells) to 10 (full spermatogenesis). Thirty seminiferous tubules from each animal were randomly examined, and the Johnsen score was assigned based on the type of the cells damaged in the seminiferous tubule. The Johnsen score was calculated by dividing the sum of all scores by the total number of seminiferous tubules examined.

### Statistical analysis

All data are presented as the means ± S.E.M. The results were analysed using the one-way analysis of variance test (One-way ANOVA) followed by Tukey’s multiple comparison test. Statistical analysis was performed using GraphPad Prism software (version 5). For all of the statistical tests, the level of significance was fixed at p<0.05.

## Results

Effect of nicorandil with or without glibenclamide co-administration on the parameters estimating CP-induced toxicity in rats

### Final body, lung and testicular weights

The toxic effect of CP on lung and testis was assessed on day 7 after 2 successive i.p. injections of CP. The CP-treated rats showed a significant reduction in final body weight and significant atrophy of the testes, which indicates the cytotoxicity and wasting effect of CP. Moreover, there was a significant increase in lung weight, which was correlated with severe lung inflammation and oedema. Treatment with nicorandil preserved the body, lung and testis weights; these results are consistent with the outcomes of the biochemical and histological examinations. By contrast, co-administration of glibenclamide, a KATP channel blocker, abolished the amelioration afforded by nicorandil treatment (Table 1).

### Testicular function

Testicular function was assessed by estimating the serum testosterone level and testicular 3β-HSD, 17β-HSD and SDH activities. The CP group showed a marked reduction in testicular function, which confirms CP-induced testicular toxicity. Treatment with nicorandil significantly increased the serum testosterone level and the testicular 3β-HSD and 17β-HSD activities. In addition, nicorandil normalized SDH activity. However, co-administration of glibenclamide completely abolished the protective effect of nicorandil on testicular function (Table 1).

### Oxidative stress markers

**Figure 1:**
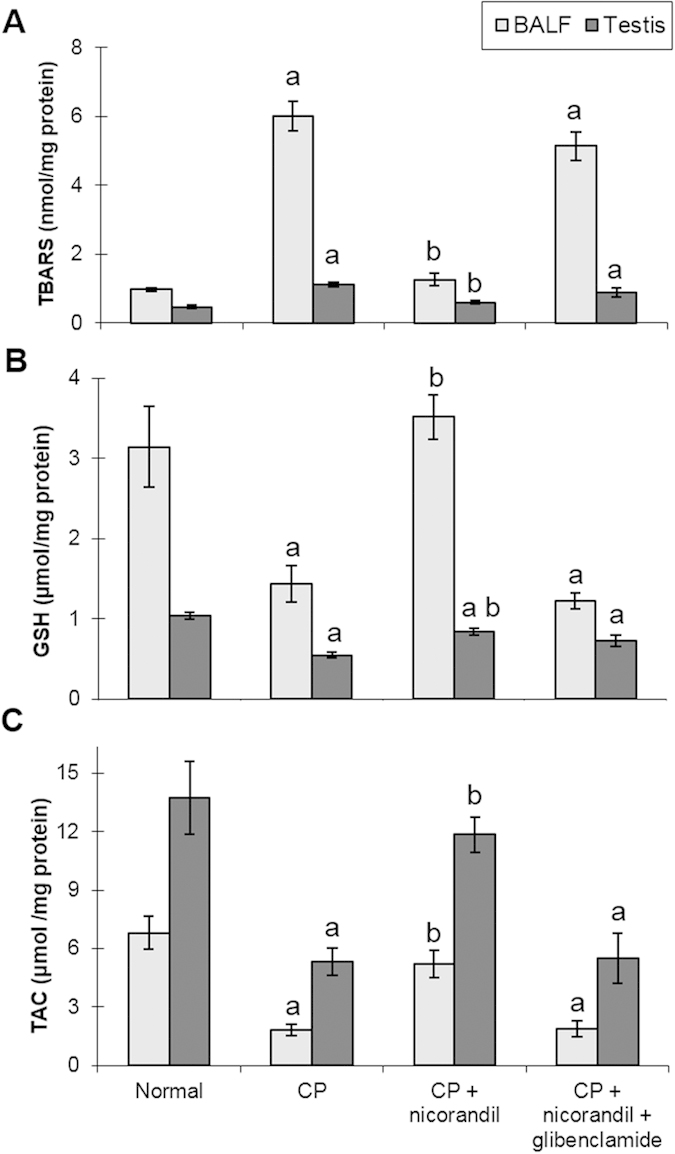
Effect of nicorandil with or without glibenclamide co-administration on oxidative stress markers in bronchoalveolar lavage fluid (BALF) and testicular tissues from rats with cyclophosphamide (CP)-induced toxicity. (**A**) Thiobarbituric acid reactive substances (TBARS). (**B**) Reduced glutathione (GSH). (**C**) Total antioxidant capacity (TAC). Each value represents the mean of 5–8 experiments ± S.E.M. ^a^*p* < *0.05 vs.* normal, ^b^*p* < *0.05 vs*. CP.

Oxidative stress status was evaluated by the measurement of TBARS as a marker of lipid peroxidation, as well as TAC and GSH as markers of enzymatic and non-enzymatic antioxidant defence systems. CP intoxication induced a state of marked oxidative stress as demonstrated by a significant increase in the TBARS level and a significant decrease in the TAC and GSH levels in BALF and testicular tissues. Pharmacological treatment with nicorandil alleviated the effect on the TBARS and GSH levels, as well as the TAC level, whereas co-administration of glibenclamide led to similar results to those of the CP-untreated group (Fig. 1).

### Inflammatory markers

**Figure 2:**
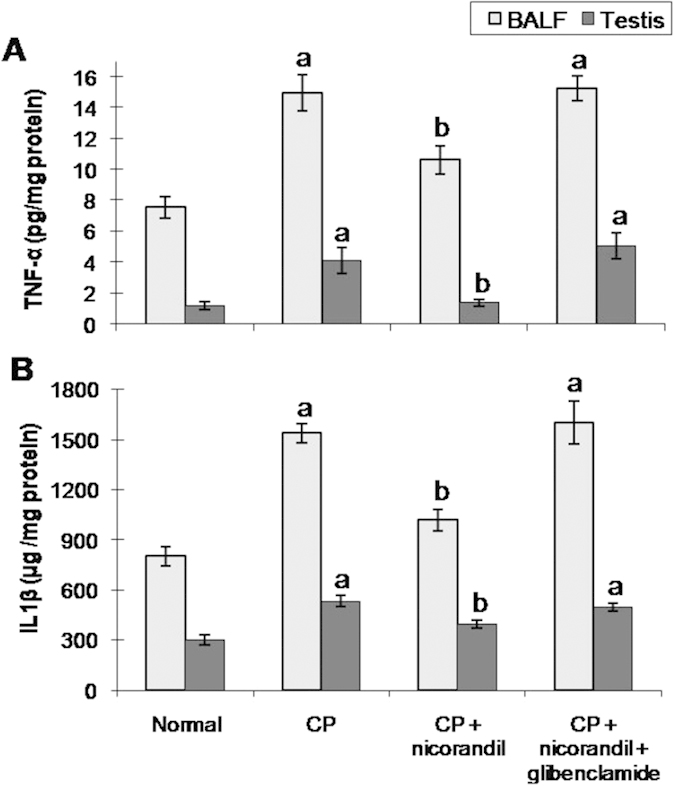
Effect of nicorandil with or without glibenclamide co-administration on inflammatory markers in bronchoalveolar lavage fluid (BALF) and testicular tissues from rats with cyclophosphamide (CP)-induced toxicity. (**A**) Tumour necrosis factor-α (TNF-α). (**B**) Interleukin-1β (IL-1β). Each value represents the mean of 5–8 experiments ± S.E.M. ^a^*p* < *0.05 vs.* normal, ^b^*p* < *0.05 vs*. CP.

Oxidative stress activates several intracellular signalling pathways leading to the upregulation of proinflammatory cytokine synthesis, including TNF-α and IL-1β. In the present study, the TNF-α and IL-1β levels were significantly elevated in the CP group in both BALF and testicular tissues, and nicorandil administration normalized these alterations in inflammatory markers. Similarly to its effect on oxidative stress markers, co-administration of glibenclamide abolished the amelioration afforded by nicorandil treatment (Fig. 2).

### NAG, histamine and LTC4

CP administration significantly elevated NAG activity in BALF. Elevation of the activity of this lysosomal enzyme is considered a potential marker of lung inflammation and fibrosis. The BALF histamine and LTC4 levels were also significantly higher in CP-treated rats, and this could be attributed to their release from mast cells sequestered in the lung during inflammatory conditions. Treatment with nicorandil normalized these abnormalities, whereas the administration of glibenclamide with nicorandil yielded results that were not significantly different from those of the CP group (Table 2).

### Fibrogenic markers

**Figure 3:**
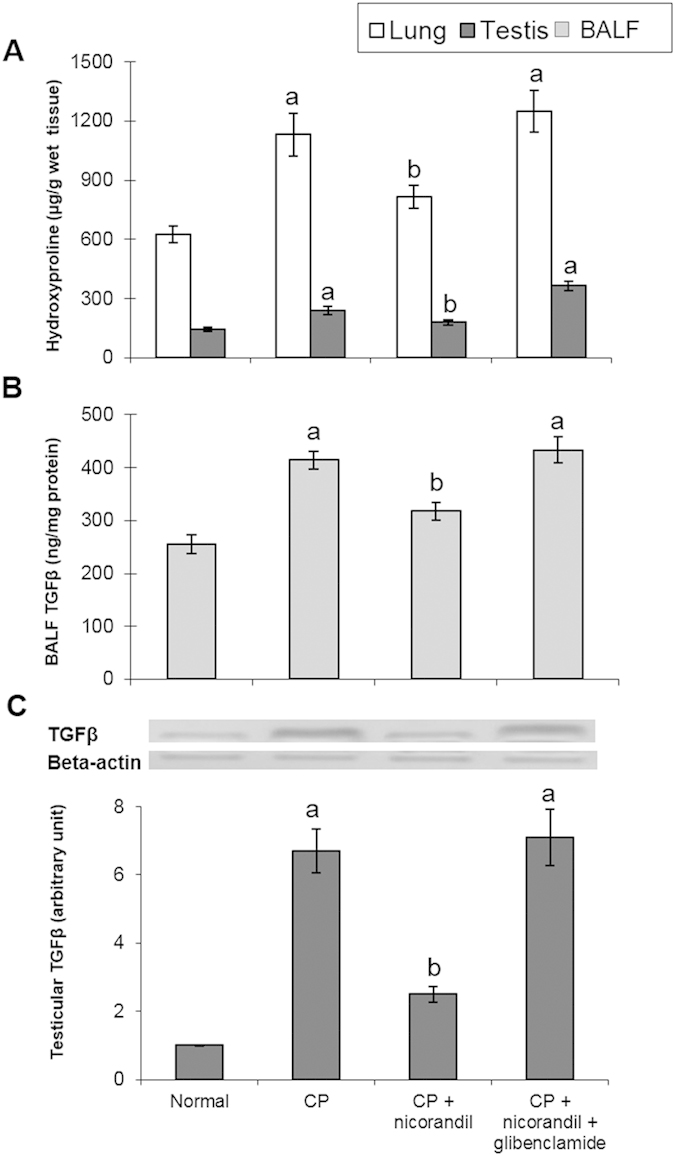
Effect of nicorandil with or without glibenclamide co-administration on fibrogenic markers in rats with cyclophosphamide (CP)-induced toxicity. (**A**) Lung and testicular hydroxyproline. (**B**) Transforming growth factor-β (TGF-β) level in bronchoalveolar lavage fluid (BALF). (**B**) Protein expression of testicular TGF-β. Each value represents the mean of 5–8 experiments ± S.E.M. ^a^*p* < *0.05 vs.* normal, ^b^*p* < *0.05 vs*. CP.

Inflammatory cytokines act as putative mediators in the chain of events leading to fibrosis. CP intoxication induced lung and testicular fibrosis, as indicated by the significant elevation of the TGF-β and hydroxyproline levels. Nicorandil significantly ameliorated this progressive state of fibrosis, as demonstrated by the normalization of the TGF-β and hydroxyproline levels. By contrast, these fibrogenic markers were still markedly elevated upon co-administration of glibenclamide, a KATP channel blocker (Fig. 3).

### Histological examinations

**Figure 4:**
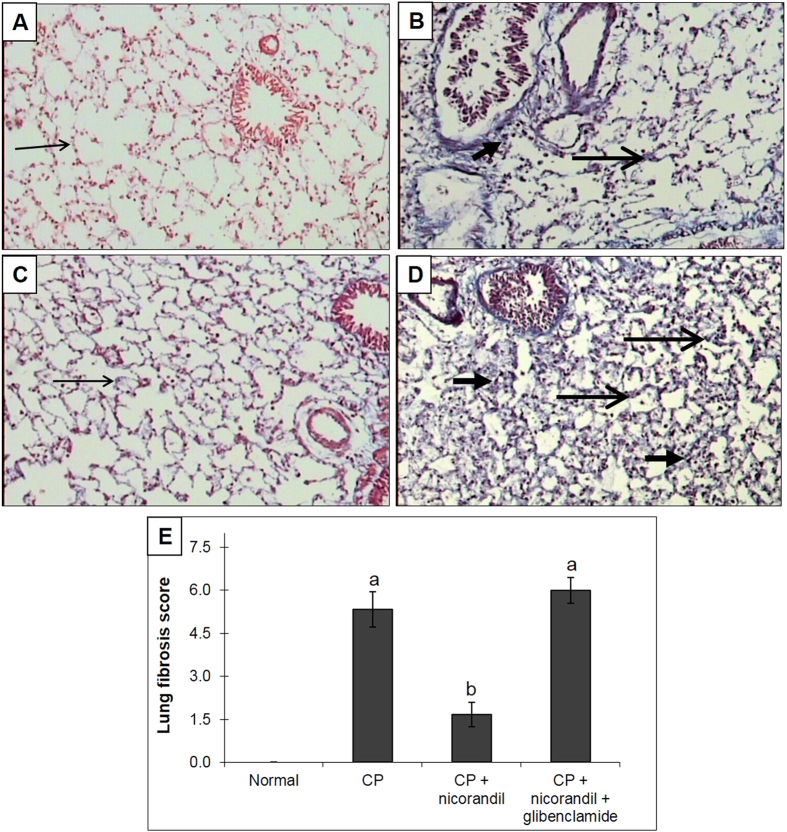
Effect of nicorandil with or without glibenclamide co-administration on histological grading of lung fibrosis in rats with cyclophosphamide (CP)-induced toxicity (Masson’s trichrome x100). (**A**) The normal group showed delicate alveoli with no fibrosis (long thin arrow), a normal number of alveolar macrophages and no inflammation. (**B**) The CP group showed an increased cellular inflammatory response with more alveolar macrophages, scattered inflammatory cells (short thick arrow), and focal alveolar damage (with alveolar and bronchiolar walls that had been thickened by fibrosis) (stained blue with Masson’s trichrome) (long thick arrow). (**C**) The nicorandil group showed minimal fibrosis of alveolar walls with unremarkable thickening (long thin arrow) and no evident inflammation. (**D**) The nicorandil + glibenclamide group showed alveolar walls thickened by fibrosis (long thick arrow) associated with an increased cellular inflammatory response (more alveolar macrophages, scattered inflammatory cells and focal oedema) (short thick arrow). (**E**) Lung fibrosis score. Each value represents the mean of 5 experiments ± S.E.M. ^a^*p* < *0.05 vs.* normal, ^b^*p* < *0.05 vs*. CP.

**Figure 5:**
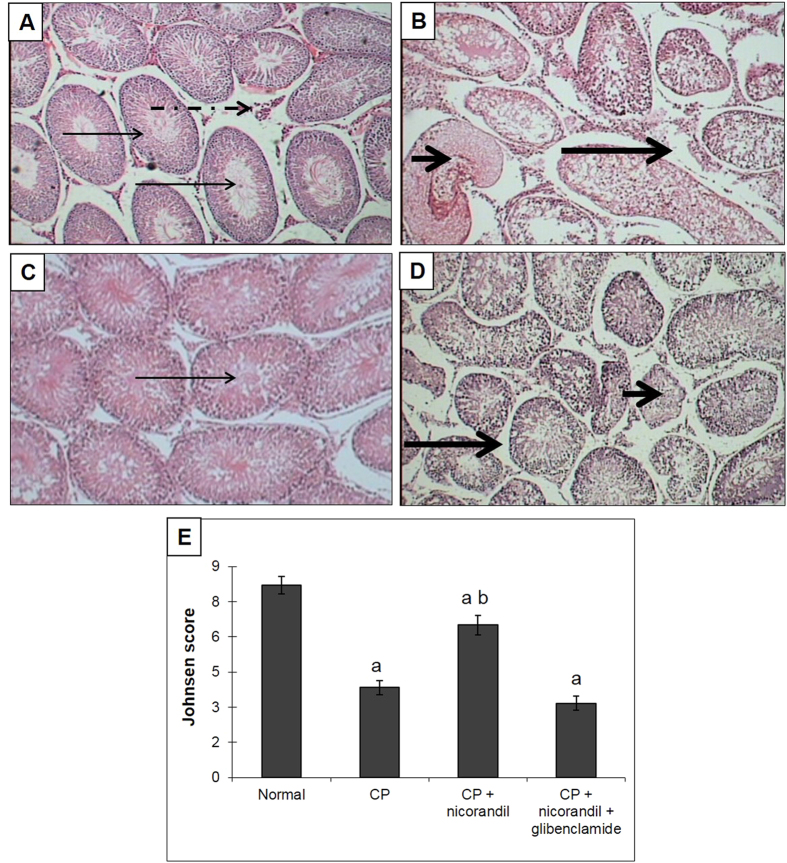
Effect of nicorandil with or without glibenclamide co-administration on the histological grading of testicular toxicity in rats with cyclophosphamide (CP)-induced testicular toxicity (H&E x100). (**A**) The normal group showed patent seminiferous tubules (long thin arrow) lined by Sertoli and spermatogenic cells with adequate Leydig cells in the interstitium (long dashed arrow). (**B**) The CP group showed tubular and germ cell injuries with no evident spermatids (short thick arrow), atrophied Leydig cells and interstitial oedema (long thick arrow). (**C**) The nicorandil group showed preserved seminiferous tubules with adequate patency and calibre (long thin arrow), as well as preserved spermatogenesis. (**D**) The nicorandil + glibenclamide group showed focal tubular injury with irregular outlines and germ cell injury (short thick arrow), with focal Leydig cell atrophy and interstitial oedema (long thick arrow). (**E**) The testicular Johnsen score. Each value represents the mean of 5 experiments ± S.E.M. ^a^*p* < *0.05 vs.* normal, ^b^*p* < *0.05 vs*. CP.

The rats in the normal group showed normal lung and testicular morphology. CP-treated animals showed significant lung damage, as demonstrated by an increased cellular inflammatory response with more alveolar macrophages, scattered inflammatory cells, and focal alveolar damage (including alveolar and bronchiolar walls thickened by fibrosis). This was confirmed by the histological grading of lung fibrosis, which showed a marked elevation of the fibrosis score in the CP group (Fig. 4). Assessments of testicular toxicity revealed that rats treated with CP exhibited low Johnsen scores, shrunken seminiferous tubules, tubular and germ cell injuries with no evident spermatids, atrophied Leydig cells and interstitial oedema (Fig. 5). Treatment with nicorandil significantly ameliorated the morphologically characteristic damage to the lungs and testes, whereas co-administration of glibenclamide abolished the protective effect of nicorandil, showing histological changes similar to the CP group.

## Discussion

CP is a widely used antineoplastic and immunosuppressive drug that causes multiple organ toxicity in humans and experimental animals. Biochemical and histological alterations induced by CP in experimental studies show similarity to those observed in humans. In this investigation, the toxic effect of CP on lung and testis was assessed on day 7 after 2 successive i.p. injections of CP. CP-treated rats showed a significant reduction in the final body and testicular weights, along with a significant increase in the lung weight. The reduction in body weight might be attributed to the cytotoxic and wasting effect of CP^32^, whereas the increase in lung weight could be due to hyperplasia, inflammation, interstitial fluid accumulation, oedema and increased collagen synthesis, as confirmed by the biochemical and histological results. Atrophy of the testis is an index of drug toxicity and was shown by a significant decrease in the serum testosterone level and testicular 3β-HSD, 17β-HSD and SDH activities. SDH activity is related to the function of germ cells, where SDH is responsible for providing energy to sperm cells by converting sorbitol to fructose^33^. The decreased activity of this enzyme after CP-induced toxicity may account for germ cell depletion in seminiferous tubules^34^. Testicular toxicity was also confirmed by histological examination, which showed shrunken seminiferous tubules, a diminished number of germ cells and atrophy of the Leydig cells in CP-treated rats. Previous studies showed that Leydig cells were more sensitive to CP and were severely affected in CP-treated animals^35^,^36^, leading to decreased testosterone synthesis and secretion.

CP intoxication induced a marked state of oxidative stress as demonstrated by a significant increase in the TBARS level and a significant decrease in the TAC and GSH levels in BALF and testicular tissues. Oxidative stress has been implicated in the pathophysiology of CP toxicity. CP and its reactive metabolite (acrolein) induce lipid peroxidation and interfere with the tissue enzymatic and non-enzymatic antioxidant defence systems^35^,^37^. The increased TBARS level observed after CP administration in this study could result in inactivation of the normal functions of enzymes, receptors and ion pumps, and could also contribute to decreased levels of cellular thiols, including GSH^10^. Moreover, the decreased TAC level could be related to inactivation of one or more sulfhydryl group residues in the antioxidant enzymes, which are essential for their catalytic activities^38^. A lack of the detoxifying enzymes aldehyde oxidase and aldehyde dehydrogenase, as well as a depletion of GSH, are the principal causes of CP’s selective toxicity for lung tissues, where ROS may injure pulmonary vascular endothelial cells, disrupt alveolar capillary membranes and allow the leakage of proteinaceous fluid into the pulmonary parenchyma^39^. Additionally, spermatozoa are more susceptible to oxidative damage than other cells because of their high concentration of polyunsaturated fatty acids and low antioxidant capacity, resulting in a decline in testosterone secretion, decreased sperm viability and increased numbers of sperm morphological defects^40^,^41^.

Oxidative stress activates several intracellular signalling pathways leading to the upregulation of proinflammatory cytokine synthesis. Our data show that the TNF-α and IL-1β levels, as well as the NAG activity, were significantly elevated in the CP group. The BALF histamine and LTC4 levels were also significantly higher in CP-treated rats. The severe inflammatory reaction could be attributed to the accumulation of inflammatory lymphocytes, neutrophils and macrophages, which have been implicated in the destruction of connective tissue through the release of proteolytic enzymes and the further release of free radicals^13^,^42^,^43^,^44^. Concerning the elevated histamine and LTC4 levels, these vasoactive chemotactic mediators are released from mast cells sequestered in the lung during inflammatory conditions and act as putative mediators in the chain of events leading to fibrosis^45^,^46^.

Furthermore, proliferative and fibrogenic responses are triggered via the development of persistent unresolved inflammation. This study showed that CP intoxication induced lung and testicular fibrosis, as indicated by significant elevation of the TGF-β and hydroxyproline levels. Tissue injury and an unresolved inflammatory response have been associated with activating fibroblasts to divide, proliferate, and synthesize collagen^47^,^48^. TGF-β has also been reported to play a critical role in the pathogenesis of fibrosis through the stimulation of collagen and fibronectin production by the activated fibroblasts, as well as through the inhibition of proteases that degrade the extracellular matrix^49^,^50^. Collagen is synthesized and secreted by fibroblasts in a soluble form and deposited extracellularly. A decrease in collagen degradation and/or an increase in its biosynthesis may be key features of the maintenance and progression of fibrosis^13^. CP-induced tissue fibrosis has been associated with an elevation of hydroxyproline content, which is characteristic of fibrosis. The histological examination of lung tissues also demonstrated an elevated grade of lung fibrosis with an extensive cellular inflammatory response, focal alveolar damage and thickened alveolar and bronchiolar walls. Finally, the histological investigation of testicular toxicity showed lower Johnsen testicular scores, atrophy and degeneration of seminiferous tubules with no evident spermatids, atrophied Leydig cells and interstitial oedema.

Treatment with nicorandil preserved the final body weight, as well as the lung and testicular weights. Moreover, treatment with nicorandil significantly increased the serum testosterone levels and testicular 3β-HSD and 17β-HSD activities and normalized SDH activity. These effects were correlated with the amelioration of biochemical and histological alterations. By contrast, glibenclamide, a KATP channel blocker, completely abolished the protective effect of nicorandil on CP-induced toxicity. Additionally, treatment with nicorandil normalized oxidative stress markers, whereas co-administration of glibenclamide gave similar results to the CP-untreated group. Nicorandil has previously been shown to reduce oxidative stress in experimental diabetic nephropathy by stimulating KATP channel opening independently of its ability to donate NO^18^. Nicorandil has also been shown to counteract the development of oxidative stress and the prostatic hyperplasia that has been related to prostatic hypoxia in spontaneously hypertensive rats^16^. The antioxidant activity of nicorandil seems to be due to its KATP channel activation, which is blocked by glibenclamide^51^. In the same manner, nicorandil inhibited the development of monocrotaline-induced pulmonary hypertension through its anti-inflammatory and anti-proliferative effects on lung tissue, which were blocked by glibenclamide^14^. Similarly to its effect on oxidative stress, the inhibitory activity of nicorandil on inflammatory cells and cytokines has been attributed to the activation of the KATP channel. In this study, nicorandil significantly reduced the TGF-β and hydroxyproline levels and ameliorated the histological changes in lung and testicular tissues, and these beneficial effects were completely blocked by glibenclamide co-administration. In support of our work, nicorandil has been shown to inhibit the activation of fibrogenic markers in experimental models of prostatic hyperplasia^16^, anti-Thy1 nephritis^15^ and Ang II-induced cardiac fibroblast proliferation^52^. These effects were attributed to its antioxidant and anti-inflammatory effects, probably mediated through KATP channel opening. The decrease in the levels of these biochemical markers in the nicorandil-treated group was strongly reflected in the histological findings in the lung and testis.

In summary, CP-induced toxicity involves a sequence of events triggered by oxidative stress and followed by inflammatory, proliferative and fibrogenic responses. Nicorandil seems to successfully prevent the development of CP-induced lung and testicular toxicity in rats, in which the activation of the KATP channel plays an important role in the protective effect of nicorandil.

## Additional Information

**How to cite this article**: Ahmed, L. A. *et al.* Role of the KATP channel in the protective effect of nicorandil on cyclophosphamide-induced lung and testicular toxicity in rats. *Sci. Rep.* **5**, 14043; doi: 10.1038/srep14043 (2015).

## Figures and Tables

**Table 1:** Effect of nicorandil with or without glibenclamide co-administration on the percentage mortality, final body weight (FBW), lung and testicular weights, and serum testosterone level, as well as the testicular 3β-hydroxysteroid (HSD), 17β-HSD and sorbitol dehydrogenase (SDH) activities, in rats with cyclophosphamide (CP)-induced toxicity.

Groups	Mortality %	FBW (g)	Lung weight (g)	Testicularweight (g)	Serumtestosterone(ng/dl)	Testicular3β-HSD(nmol NADH/min/mg protein)	Testicular17β-HSD(nmol NADPH/min/mg protein)	TesticularSDH(U/mg protein)
Normal	0%	201.70 ± 2.00	2.01 ± 0.12	1.97 ± 0.11	180.25 ± 4.56	4.98 ± 0.38	6.71 ± 0.39	5.02 ± 0.41
CP	30%	150.71 ± 4.70^a^	3.80 ± 0.21^a^	0.91 ± 0.10^a^	45.75 ± 5.67^a^	2.09 ± 0.17^a^	3.33 ± 0.40^a^	2.98 ± 0.29^a^
CP + nicorandil	0%	190.80 ± 6.88^b^	1.82 ± 0.24^b^	1.72 ± 0.11^b^	135.50 ± 9.04^ab^	3.61 ± 0.34^ab^	5.03 ± 0.41^ab^	4.40 ± 0.39^b^
CP + nicorandil + glibenclamide	50%	143.82 ± 4.21^a^	3.70 ± 0.36^a^	1.01 ± 0.17^a^	29.04 ± 3.70^a^	1.59 ± 0.27^a^	2.22 ± 0.27^a^	2.19 ± 0.18^a^

Each value represents the mean of 5–8 experiments ± S.E.M. ^a^*p* < *0.05 vs.* normal, ^b^*p*< * 0.05 vs*. CP.

**Table 2:** Effect of nicorandil with or without glibenclamide co-administration on *N-*acetyl-β-D-glucosaminidase (NAG), histamine and leukotriene C4 (LTC4) levels in bronchoalveolar lavage fluid (BALF) from rats with cyclophosphamide (CP)-induced toxicity.

Groups	NAG(μmol p-nitrophenol/min/mg protein)	Histamine(μg/mg protein)	LTC4(μg/mg protein)
Normal	15.84 ± 1.32	77.79 ± 8.31	3.18 ± 0.27
CP	33.54 ± 2.01 a	159.63 ± 8.91 a	9.45 ± 0.72 a
CP + nicorandil	23.67 ± 1.68 ab	68.85 ± 6.36 b	5.28 ± 0.63 b
CP + nicorandil + glibenclamide	26.97 ± 1.86 a	174.81 ± 10.83 a	11.88 ± 0.51 a

Each value represents the mean of 5–8 experiments ± S.E.M. ^a^*p* < *0.05 vs.* normal, ^b^*p* < *0.05 vs*. CP.
